# Active Vibration Avoidance Method for Variable Speed Welding in Robotic Friction Stir Welding Based on Constant Heat Input

**DOI:** 10.3390/ma17112593

**Published:** 2024-05-28

**Authors:** Guanchen Zong, Cunfeng Kang, Shujun Chen

**Affiliations:** College of Mechanical and Energy Engineering, Beijing University of Technology, Beijing 100124, China; zongguanchen@emails.bjut.edu.cn (G.Z.); sjchen@bjut.edu.cn (S.C.)

**Keywords:** RFSW, vibration analysis, modal analysis, vibration feedback, robot dynamics

## Abstract

Robotic Friction Stir Welding (RFSW) technology integrates the advantages of friction stir welding and industrial robots, finding extensive applications and research in aerospace, shipbuilding, and new energy vehicles. However, the high-speed rotational process of friction stir welding combined with the low stiffness characteristics of serial industrial robots inevitably introduces vibrations during the welding process. This paper investigates the vibration patterns and impacts during the RFSW process and proposes an active vibration avoidance control method for variable speed welding based on constant heat input. This method utilizes a vibration feedback strategy that adjusts the spindle speed actively if the end-effector’s vibration exceeds a threshold, thereby avoiding the modal frequencies of the robot at its current pose. Concurrently, it calculates and adjusts the welding speed of the robot according to the thermal equilibrium equation to maintain constant heat input. A simplified dynamic model of the RFSW robot was established, and the feasibility of this method was validated through simulation experiments. This study fills the gap in vibration analysis of RFSW and provides new insights into control strategies and process optimization for robotic friction stir welding.

## 1. Introduction

Friction Stir Welding (FSW) technology, invented in 1991, is a solid-state joining technique [1]. Traditional arc welding is prone to the formation of brittle phases and cracks during the cooling process due to its high temperature [2]. FSW is characterized by low welding temperatures and minimal residual stresses, which have led to its widespread application in aerospace, automotive manufacturing, and shipbuilding industries [3]. FSW technology not only performs excellently in the welding of similar metals but also demonstrates irreplaceable advantages in the welding of dissimilar metals [4]. Traditional FSW equipment, such as gantry and bench types, can perform two-dimensional planar welding; however, they are less effective for complex spatial curve welding tasks [5]. To meet the requirements for greener, lighter, and more flexible development, Robotic Friction Stir Welding (RFSW) has emerged [6]. Nonetheless, the relatively low stiffness of serial industrial robots can lead to deviations and vibrations at the end-effector during the welding, thereby imposing higher demands on the control and optimization of the robotic systems [7,8].

Research on control and optimization methods for RFSW primarily focuses on reducing end-effector deviation to enhance welding precision [9,10,11]. Xiao et al. [12] proposed a constant plunge depth control method for RFSW based on online trajectory generation, achieving compensation for the plunge depth error of the stirring tool during welding. However, studies on the effects and optimization of vibrations during the RFSW process are limited. Concerning the impact of vibrations on the FSW process, some scholars have conducted relevant research. Notably, Friction Stir Vibration Welding (FSVW) and Ultrasonic-Assisted Friction Stir Welding (UAFSW) are representative techniques. FSVW involves applying vibrations to the worktable during the welding to improve weld quality [13]. Bagheri et al. [14] investigated the mechanical behavior and microstructure of AA6061-T6 joints manufactured by the FSVW technique. The results indicated that, compared to FSW, the FSVW technique could significantly enhance the strength and hardness of the joints. Abbasi et al. [15] explored the effects of vibration on dynamic recrystallization during the magnesium alloy FSVW process. The results suggested that compared to traditional FSW, vibration could promote nucleation and grain growth, improve grain refinement, and thus affect the microstructure and mechanical properties of the weld zone. UAFSW employs an auxiliary device that applies ultrasonic vibrations to the weld zone during the welding process, thereby increasing the energy input and improving weld quality [16]. Wu et al. [17] investigated the effects of ultrasonic vibrations on the FSW process of Al/Mg alloys. The experimental results indicated that ultrasonic vibrations significantly enhanced the flowability of the material in the weld zone, improving the microstructure and mechanical properties of the welds. Tian et al. [18] explored the role of ultrasonic vibrations in Al/Cu joints. Their studies found that ultrasonic vibrations could reduce tool torque and axial force, enhance material flow and homogeneity, and significantly improve mechanical properties.

In RFSW processes without external devices, vibrations primarily arise due to the welding forces involved. Research suggests that periodic forces during the welding process are caused by the eccentric error of the stirring pin relative to the spindle [19]. This eccentricity results from misalignment of the stirring tool during installation and the wear-induced internal clearance in the spindle under high load conditions. These are inherent characteristics of the FSW and cannot be completely eliminated [20]. Some researchers have found that moderate eccentric errors can facilitate material flow and enhance weld quality. However, excessive eccentric errors can increase fluctuations in welding forces, degrade weld quality, and lead to more welding defects [21,22]. Traditional FSW equipment, which has higher stiffness, is less affected by changes in welding forces [23]. In contrast, the lower stiffness at the robot end-effector in FSW robots leads to forced vibrations under the influence of welding forces, thereby affecting the welding process and the quality of the welds.

Similar to RFSW, robotic milling also uses general industrial robots as the work platform. Scholars have conducted extensive research on vibrations during the robotic milling process. The studies mainly focus on vibration detection and signal analysis [24], optimization of robot poses [25], active vibration suppression [26], and structural optimization of robots [27]. These studies provide important references for vibration analysis and optimization in RFSW. Among them, experimental modal analysis is an effective method for studying the dynamic characteristics of robots. Chen et al. [28] developed a method to rapidly predict the frequency response function at the tool tip of industrial robots in various poses based on hammer test data from a series of predefined positions. Nguyen et al. [29] predicted modal parameters at the robot tool tip using a Gaussian process regression model based on impact hammer tests at the robot end-effector, and these parameters were used to predict vibrations at the tool tip during the robotic milling process. Conducting modal analysis on the FSW robot can reveal its dynamic characteristics, thereby allowing for targeted optimization of process parameters and the development of optimized control methods.

The structural characteristics of serial industrial robots, combined with the processing method of high-speed rotation in FSW, inevitably lead to vibration during the welding process. Currently, there is limited research on the vibration phenomenon and its effects in RFSW. Studying the impact of vibration on the welding process and quality, the characteristics of vibration during welding, and optimization methods for welding vibration are of great significance for improving the welding quality and efficiency of RFSW. This study experimentally investigated the vibration phenomenon and its effects during RFSW. Based on the experimental results and the dynamic characteristics of FSW robots, a vibration avoidance method based on constant heat input and variable spindle speed welding was proposed and simulated. This study fills the gap in the analysis and optimization of vibration in RFSW, providing new insights for control strategies and process optimization in RFSW. The rest of this study is organized as follows: Section 2 introduces the methods and equipment for welding vibration experiments and modal analysis experiments, active vibration avoidance method for variable-speed welding, constant heat input control method, and simplified model of robot end-effector dynamics. Section 3 analyzes and discusses the experimental results, and simulates and verifies the active vibration avoidance method for variable-speed welding based on constant heat input. Section 4 summarizes the research.

## 2. Methodology and Materials

### 2.1. Experimental Equipment and Procedures

**Figure 1:**
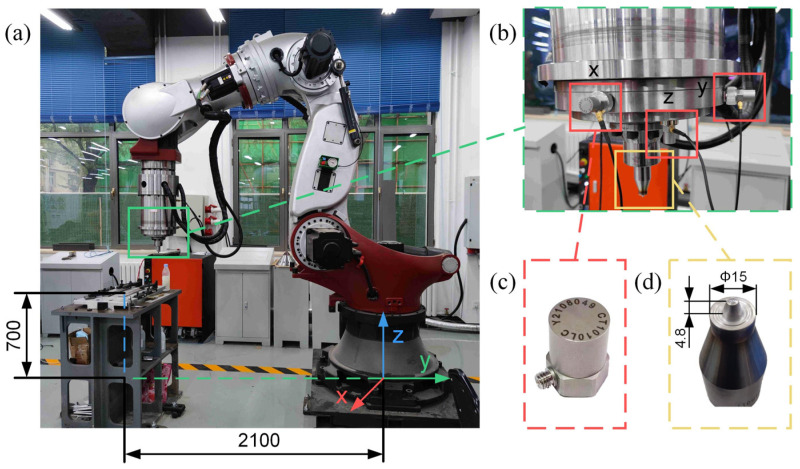
The experiment equipment: (**a**) FSW robot and workbench; (**b**) sensors location; (**c**) acceleration vibration sensor; (**d**) conical stirring tool.

According to existing studies, the eccentric error (EE) of the stirring tool causes periodic variations in welding forces and cannot be completely eliminated [19,20]. To explore the vibration conditions under different parameters during the RFSW process and their impact on weld quality, this study modified the eccentric error of the stirring tool by grinding its handle. Initially, the study examined the end-effector vibrations of the robot under various eccentric errors and spindle speeds. Subsequently, the mechanical properties of the welds under different vibration conditions were investigated. Then, the modal parameters of the robot were analyzed using experimental modal analysis methods. The experimental equipment is as follows:

Figure 1a shows the relative position of the FSW robot and the welding workbench. The FSW robot is a ZK-500 heavy-duty robot equipped with a six-axis force sensor and a specialized spindle for FSW, produced by Zhen Kang Machinery Co., Ltd., Nantong, China. An accelerometer for vibration detection is mounted at the end of the robot, as shown in Figure 1b, produced by CHENGTEC Electronics Co., Ltd., Shanghai, China. The welding experiments used AA6061-T6 aluminum alloy plates measuring 300 × 150 × 5 mm. The stirring tool used was a 5 mm conical pin, as shown in Figure 1d. The material of the stirring tool is H13 steel, with a shoulder diameter of 15 mm, a stirring pin tip diameter of 3.5 mm, a bottom diameter of 7 mm, and a length of 4.8 mm. During the welding process, the plunge speed is 2 mm/s, and the dwell time varies from 5 to 10 s depending on the spindle speed.

**Figure 2:**
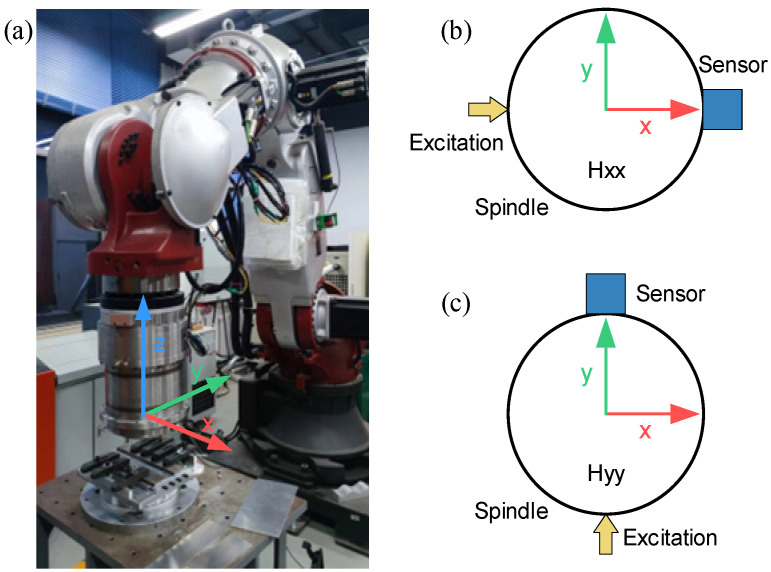
Modal analysis of FSW robot: (**a**) robot working position; (**b**,**c**) hammering test position.

This study employs experimental modal analysis using hammering tests to analyze the modal parameters of the robot’s end-effector near its working position. Initially, the robot’s end-effector was positioned above the worktable, aligning the spindle axis perpendicular to the workbench as shown in Figure 2a. Subsequently, vibration sensors were attached to the robot’s end-effector, and hammering tests were conducted in the x and y directions using a force hammer, as illustrated in Figure 2b,c. Then, we recorded the vibration response signal.

### 2.2. Optimization Methods

#### 2.2.1. Active Variable Speed Vibration Avoidance Method with Feedback Control

Vibration feedback control is a real-time control method that involves online measurement of vibrations from the actuator and adjusts operational parameters based on the current state of the system [30]. Vibration signals are easy to collect and are minimally susceptible to interference. Vibration sensors are easy to install and have little impact on the system structure, thus offering significant advantages in system stability control [31].

During the RFSW process, the frequency of the vibration signals generated by welding is solely dependent on the spindle speed [32]. When the spindle rotational frequency approaches a natural frequency of the robot at its current pose, the vibration intensity significantly increases. By installing an accelerometer at the robot’s end-effector, vibrations at the end can be monitored throughout the welding process. Then, based on changes in the intensity of the vibration signals, the spindle speed can be adjusted in real time. This adjustment alters the vibration frequency during the welding process, thereby avoiding resonance.

**Figure 3:**
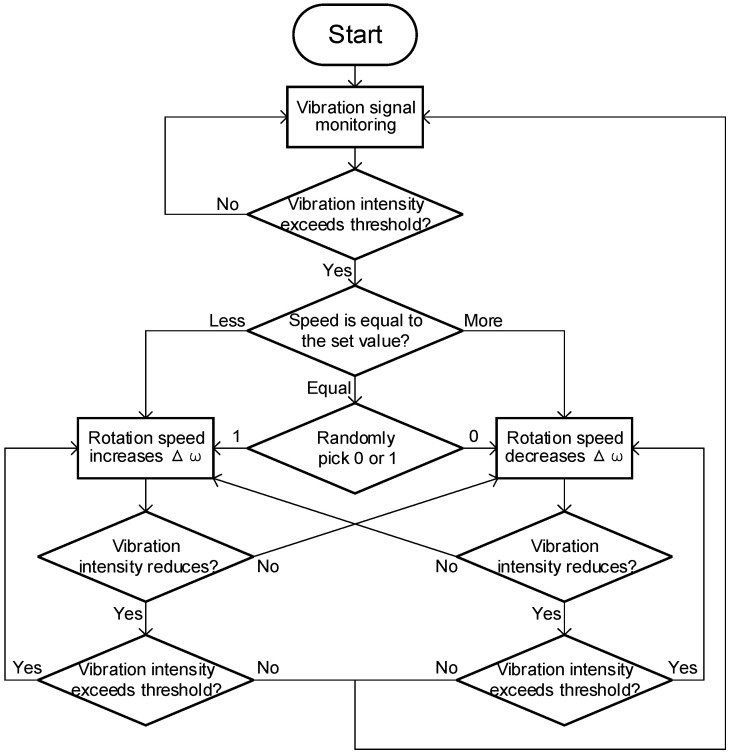
Vibration feedback control flow chart.

The vibration feedback control strategy is as follows: Initially, during the welding process, a vibration sensor installed at the robot’s end-effector monitors the intensity of vibration signals. If an increase in vibration intensity that exceeds a certain threshold is detected, the control system adjusts the spindle speed as follows: if the current speed is less than the set speed, the speed is increased by Δω; if the current speed is greater than the set speed, it is decreased by Δω; if the speed is equal to the set speed, the speed is randomly increased or decreased by Δω. After a speed adjustment, if the vibration signal intensity decreases, the system checks whether the intensity still exceeds the threshold. If it does not, the current speed is maintained, and the system returns to monitoring the spindle speed; if the intensity still exceeds the threshold, the speed is further adjusted. If, however, the vibration signal intensity increases following a speed change, the opposite adjustment is made, and the vibration threshold is reassessed. The control flowchart is shown as Figure 3.

#### 2.2.2. Constant Heat Input Control

The temperature distribution during the FSW process is one of the key factors that determine weld quality, as it directly affects the microstructure within the weld and ultimately influences the mechanical properties of the joint. It is influenced by welding parameters such as spindle speed, welding speed, and downward force. The heat generation in the FSW process is primarily composed of three parts: the frictional heat generated by the shoulder against the surface of the workpiece, the frictional heat between the stirring pin and the internal base material, and the heat produced by the plastic deformation of the metal, as shown in Equation (1) [33].

(1) $$Q_{\mathrm{heat}}=Q_{\mathrm{shoulder}}+Q_{\mathrm{pin}}+Q_{\mathrm{plastic}}$$

Assuming the use of a conical stirring tool, the formulas for calculating the heat generation power for each part are as follows:

Frictional heat power from the stirring tool shoulder [33]:

(2) $$W_{\mathrm{shoulder}}=\omega M_{\mathrm{shoulder}}=\omega \int_{R_{2}}^{R_{1}}dM=\frac{2\pi \omega \mu p}{3}\left(R_{1}^{3}-R_{2}^{3}\right)$$

where ω is spindle speed; $M_{\mathrm{shoulder}}$ is the torque at the shoulder; μ is the friction coefficient; P is the axial pressure; $R_{1}$ is the shoulder radius; $R_{2}$ is the root radius of the stirring tool.

Frictional heat power from the stirring pin [33]:

(3) $$W_{\mathrm{pin}}=\omega M_{\mathrm{pin}}=\omega \int_{0}^{H}2\pi \mu p{\left(R_{3}+h\tan\alpha \right)}^{2}\frac{dh}{\cos\alpha }=\frac{2\pi \omega \mu p}{3\sin\alpha }\left(R_{2}^{3}-R_{3}^{3}\right)$$

where $M_{\mathrm{pin}}$ is the torque at the stirring pin; H is the length of the stirring pin; α is the semi-apex angle of the stirring pin; $R_{3}$ is the radius at the end of the stirring pin.

Metal plastic deformation heat generation power [34]:

(4) $$W_{\;\mathrm{plastic}\;}={\xi \sigma }_{e}\dot{\varepsilon }$$

where ξ is the thermal efficiency of plastic deformation; $\sigma_{e}$ is the equivalent stress; $\dot{\varepsilon }$ is the plastic strain rate.

The plastic strain rate $\dot{\varepsilon }$ can be expressed as [35]:

(5) $$\dot{\varepsilon }=\frac{1}{L_{e}}R_{m}\cdot 2\pi \cdot r_{e}$$

where $r_{e}$ is the effective radius of the recrystallization zone; $L_{e}$ is the effective depth of the recrystallization zone; $R_{m}$ is the average material flow rate.

During the FSW process, if the vibration sensor detects a vibration signal that exceeds a threshold, the spindle speed is altered under the vibration feedback control system to avoid the natural frequencies of the robot at its current pose. Following the change in spindle speed, the heat generation power during the welding process also changes.

To ensure stability during the welding process, this study adjusts the welding speed to keep the heat input per unit area constant, minimizing the impact of changes in spindle speed on weld quality.

Assuming the total heat generation power at the stirring tool before adjustment is $W_{1}$ with a welding speed of $v_{1}$, and after adjustment, the total heat generation power is $W_{2}$ with a welding speed of $v_{2}$, to maintain constant heat input per unit area, the heat input balance equation is established as shown in Equation (6).

(6) $$\frac{W_{1}}{v_{1}}=\frac{W_{2}}{v_{2}}$$

Based on Equation (6), by substituting the spindle speeds before and after adjustment and the welding speed before adjustment into the formulas, the welding speed after adjustment can be calculated.

### 2.3. Simulation Models

**Figure 4:**
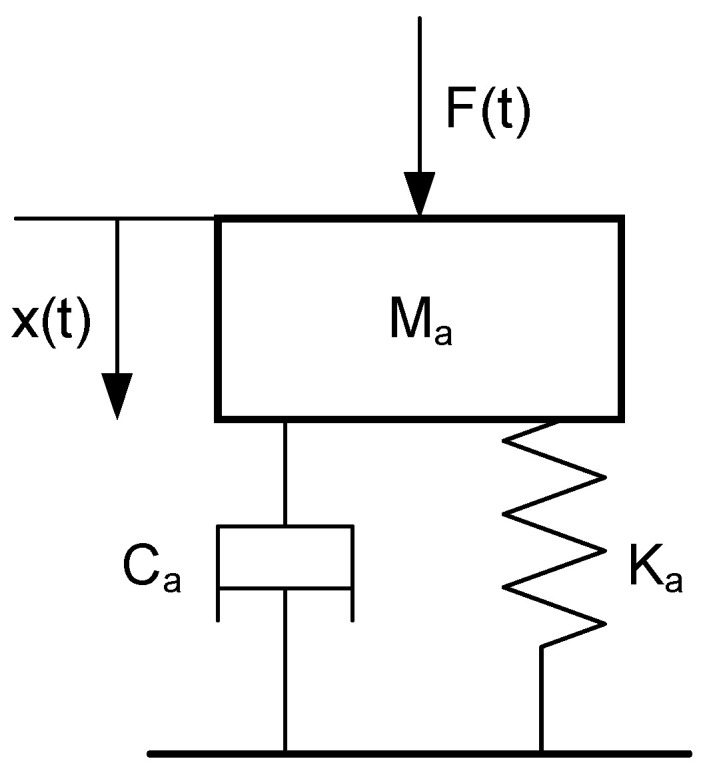
Simplified model of robot end.

To adequately reflect the changes in vibration intensity at the robot’s end-effector with varying robot poses, this paper simplifies the robot’s dynamic model and considers the robot’s end-effector as a single-degree-of-freedom spring-damper mass model, as shown in Figure 4.

Based on the simplified model of the FSW robot’s end-effector shown in Figure 4, a simplified dynamic model of forced vibration during the welding process is established as follows [31]:

(7) $$M_{a}\ddot{x}\left(t\right)+C_{a}\dot{x}\left(t\right)+K_{a}x\left(t\right)=F\left(t\right)$$

where $M_{a}$ is the equivalent mass of the system; $C_{a}$ is the damping coefficient of the system along the welding direction; $K_{a}$ is the stiffness of the system along the welding direction; F(t) is the welding force experienced by the robot’s end in the welding direction.

F(t) can be decomposed into the sum of a constant force and a periodic force, as shown in Equation (8).

(8) $$F\left(t\right)=F_{0}+F_{v}\sin\left(\omega t\right)$$

where $F_{0}$ is the constant force exerted on the stirring tool in the welding direction during the welding process; $F_{v}$ is the amplitude of the periodic force exerted on the stirring tool in the welding direction; ω is the spindle speed.

In analyzing the vibration conditions during the FSW process, only the scenario when the system reaches a steady state during the welding process is considered. Hence, the transient part of the vibration response and the impact of the constant force on the system are ignored. The steady-state solution of the equation is derived from Equation (7) as follows:

(9) $$x_{s}\left(t\right)=X_{s}\sin\left(\omega t-\varphi \right)$$

where $X_{s}$ is the amplitude of the steady-state response; φ is the phase difference of the steady-state response.

The damping ratio ξ, frequency ratio λ, and natural frequency $\omega_{n}$ are defined as follows:

(10) $$\xi =\frac{C_{a}}{2\sqrt{M_{a}K_{a}}},\lambda =\frac{\omega }{\omega_{n}},\omega_{n}=\sqrt{\frac{K_{a}}{M_{a}}}$$

The following can be obtained:

(11) $$X_{s}=\frac{F_{v}}{K_{a}\sqrt{{\left(1-\lambda^{2}\right)}^{2}+{\left(2\lambda \xi \right)}^{2}}},\varphi =\tan^{-1}\frac{2\lambda \xi }{1-\lambda^{2}}$$

The equivalent mass of the system $M_{a}$ can be approximated using Equation (12) [36].

(12) $$M_{a}=\frac{\left||K_{c}\right||}{\omega_{n}}$$

where $\left||K_{c}\right||$ is the Euclidean norm of the Cartesian stiffness at the robot’s end-effector.

Ignoring the internal damping of the robot, the damping coefficient $C_{a}$ can be expressed as follows:

(13) $$C_{a}=\frac{F_{C_{a}}}{v}$$

where $F_{C_{a}}$ is the resistance experienced by the stirring tool; v is the speed of the stirring tool’s movement.

The stiffness along the welding direction $K_{a}$ and the Cartesian stiffness $K_{c}$ at the robot’s end-effector can be calculated using the joint stiffness data shown in Table 1, combined with the robot’s pose [37]. Since the dynamic model simulated the vibration conditions of the FSW robot near its working position, the calculations were performed using the joint angles at the working pose, which are (−83.60°, 22.12°, 10.37°, 0°, 57.51°, −83.60°).

## 3. Results and Discussion

### 3.1. Vibration Analysis and Impact in RFSW

#### 3.1.1. Results of Vibration Conditions under Different Eccentric Errors and Spindle Speeds

**Figure 5:**
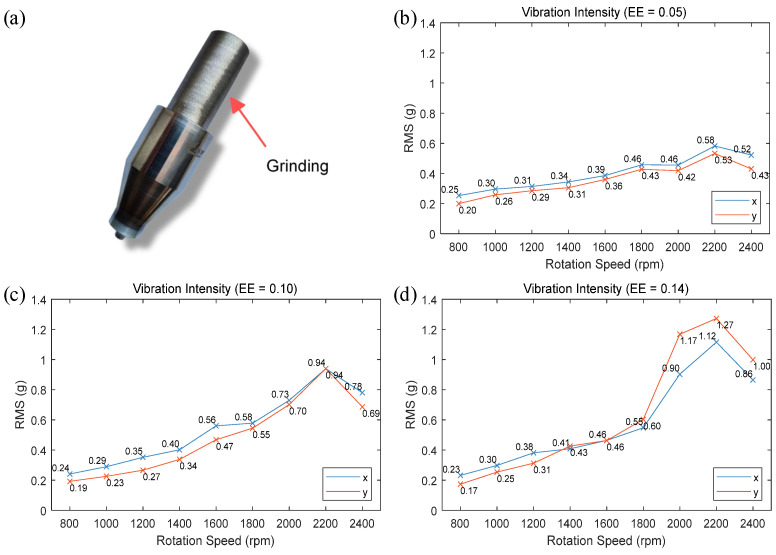
Vibration intensity experiments with different eccentric errors: (**a**) grinding position of stirring tool handle; (**b**–**d**) vibration intensity with different eccentric errors.

To investigate the effects of different stirring tool eccentric errors and spindle speeds on the vibrations during welding, the handle of the stirring tool, as shown in Figure 5a, was ground to achieve different eccentric errors. The initial eccentricity error of the stirring tool is 0.05 mm. According to existing research, an eccentricity error within 0.2 mm is conducive to improving welding quality [21]. Based on this, the eccentricity error of the stirring tool after grinding were 0.05 mm, 0.10 mm, and 0.14 mm. Welding experiments were conducted using these tools along the +y direction. The experimental parameters were as follows: spindle speeds ranging from 800 rpm to 2400 rpm in 200 rpm increments, a welding speed of 2 mm/s, and a welding tilt angle of 2 degrees. Vibration signals in the x and y directions were recorded during welding. The root mean square (RMS) of the vibration signals was used as the criterion for assessing vibration intensity [38]. The calculation method of RMS is shown in Equation (14) [38]. Vibration data for approximately 10 s during the stable welding phase of welding were captured, and the curves illustrating the variation of end-effector vibration intensity with spindle speed under different eccentric errors are depicted in Figure 5b–d. The blue curves represent the vibration intensity of the robot end-effector in the x-direction of the base coordinate system, while the red curves represent the vibration intensity of the robot end-effector in the y-direction of the base coordinate system.

(14) $$\mathrm{RMS}=\sqrt{\frac{1}{T}\int_{0}^{T}{\left[f\left(t\right)\right]}^{2}dt}$$

The figures indicate that when the spindle speed is below 1800 rpm, the vibration intensity during the welding process exhibits a nearly linear relationship with increases in spindle speed. Above 1800 rpm, the vibration intensity fluctuates more noticeably as the spindle speed continues to rise. In all three experimental groups, the vibration intensity peaks at 2200 rpm and then diminishes, suggesting the presence of a natural frequency near 36.67 Hz (2200 rpm) under the current pose of the FSW robot. With smaller eccentric errors of the stirring tool, the increase in end-effector vibration intensity is less pronounced and more uniform across changes in spindle speeds. As the eccentric error increases, the magnitude of vibration intensity also increases with spindle speeds. When the spindle rotational frequency approaches the natural frequency of the FSW robot, the impact of eccentric error on vibration intensity sharply increases.

The experimental results summarize that the eccentric error of the stirring tool is a significant factor affecting the end-effector vibrations of the FSW robot. When the spindle rotational frequency is close to the robot’s natural frequency, excessive eccentric error can cause severe vibrations at the robot’s end-effector.

#### 3.1.2. Results of Mechanical Properties of Welds under Different Vibration Conditions

The experiments indicate that an increase in the eccentric error of the stirring tool generates additional vibrations at the end-effector of the FSW robot. Existing research suggests that appropriately increasing the eccentric error of the stirring tool and applying vibrations during the welding process can enhance weld quality and increase joint strength [13,21,22]. Therefore, investigating the impact of vibrations on the mechanical properties of the welds during the RFSW process is highly valuable for establishing process standards and optimizing control strategies.

**Figure 6:**
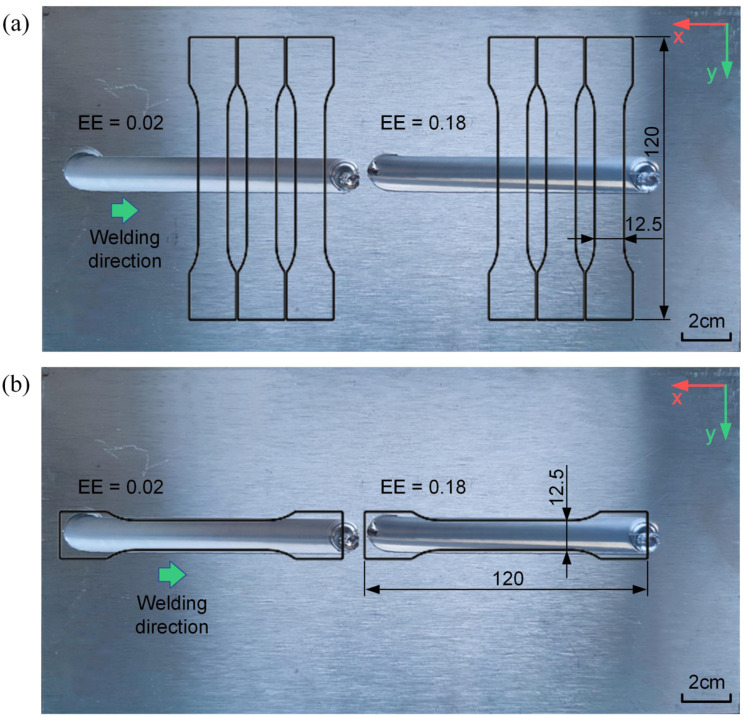
Welding experiments and tensile specimens: (**a**) transverse tensile specimens; (**b**) longitudinal tensile specimens.

**Figure 7:**
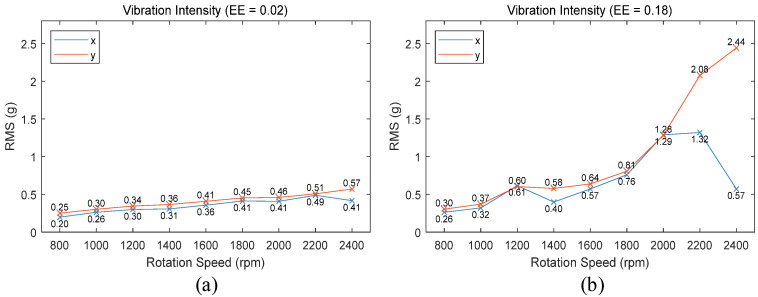
Vibration intensity with different eccentric errors: (**a**) eccentric errors = 0.02; (**b**) eccentric errors = 0.18.

This experiment conducted welding tests using conical threaded stirring tools with eccentric errors of 0.02 mm and 0.18 mm on the plates shown in Figure 6, welding along the −x direction. The spindle speeds were set between 800 rpm and 2400 rpm in 200 rpm increments, with a welding speed of 2 mm/s and a welding tilt angle of 2 degrees. Vibration signals in the x and y directions were recorded during welding. The RMS of the vibration signals served as the criterion for assessing vibration intensity. Vibration data for approximately 10 s during the stable welding stage of welding were captured, and the curves depicting the change in end-effector vibration intensity with varying spindle speeds under different eccentric errors are shown in Figure 7.

From Figure 7, it is evident that when the eccentric error of the stirring tool is small, the vibration intensity at the robot’s end-effector is similar to the data shown in Figure 5b. With a larger eccentric error, there are significant differences in vibration intensity changes. As shown in Figure 7b, when the spindle speed reaches 1200 rpm, there is a notable increase in vibration intensity. At spindle speeds above 1800 rpm, the vibration intensity in the x-direction peaks at 2200 rpm and then decreases, whereas in the y-direction, the vibration intensity continues to rise rapidly after 2000 rpm, creating a significant disparity with the x-direction. This indicates that compared to welding in the +y direction, when welding in the −x direction, there exists a natural frequency near 20 Hz (1200 rpm) under the current pose of the FSW robot, and the natural frequencies in the x and y directions are different.

**Figure 8:**
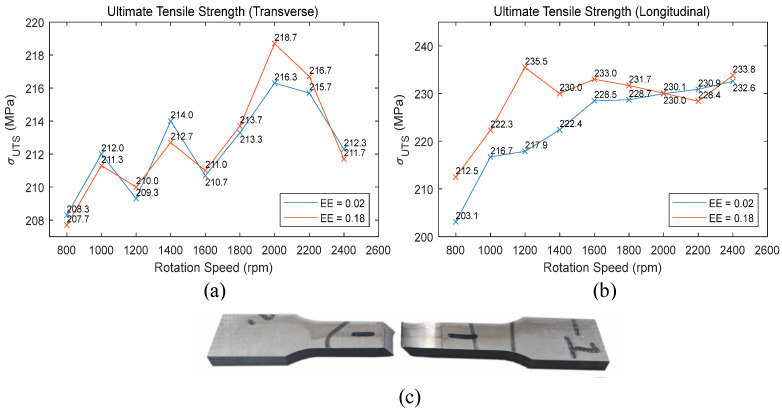
Tensile test results: (**a**) ultimate tensile strength (transverse); (**b**) ultimate tensile strength (longitudinal); (**c**) transverse tensile specimen fracture location.

As shown in Figure 6, referencing the ASTM-E8 standard, transverse and longitudinal tensile test specimens were prepared from the weld zones and subjected to tensile testing. The curves illustrating the variation of tensile strength with spindle speed under different stirring tool eccentric errors are depicted in Figure 8. The tensile strength data is the result obtained after averaging three experimental trials.

From Figure 8a, it is observed that the transverse tensile strength of the weld increases with the spindle speed, reaching a maximum at 2000 rpm before starting to decline. The eccentric error of the stirring tool has a minor impact on the transverse tensile strength. Observing the fracture locations of the tensile specimens as shown in Figure 8c, transverse fractures primarily occur in the heat-affected zone on the advancing or retreating side. During the FSW process of 6061 aluminum alloy, this region is affected by thermal cycles, which reduce material strength and commonly result in fractures at these locations during tensile testing [39].

Compared to transverse tensile tests, longitudinal tensile tests more accurately reflect the mechanical properties of the weld area. As shown in Figure 8b, with a lower eccentric error of the stirring tool, the longitudinal tensile strength of the weld gradually increases as the spindle speed increases. There is a significant increase in strength from 800 rpm to 1600 rpm, and the increase flattens out above 1600 rpm. With a larger eccentric error, except at 2200 rpm, there is a general increase in tensile strength. In the mid to low spindle speed range from 800 to 1600 rpm, the increase in strength is more pronounced, with the greatest increase occurring at 1200 rpm. Analyzing this in conjunction with the vibration intensity during the welding process shown in Figure 6, it is found that at 1200 rpm, the increase in longitudinal tensile strength correlates with the increase in vibration intensity. Above 1800 rpm, the increase in vibration intensity has a smaller impact on tensile strength and even causes a reduction in strength. Therefore, in the mid to low spindle speed range, increasing the eccentric error of the stirring tool to enhance the vibration intensity during the welding process can improve the mechanical properties of the weld.

**Figure 9:**
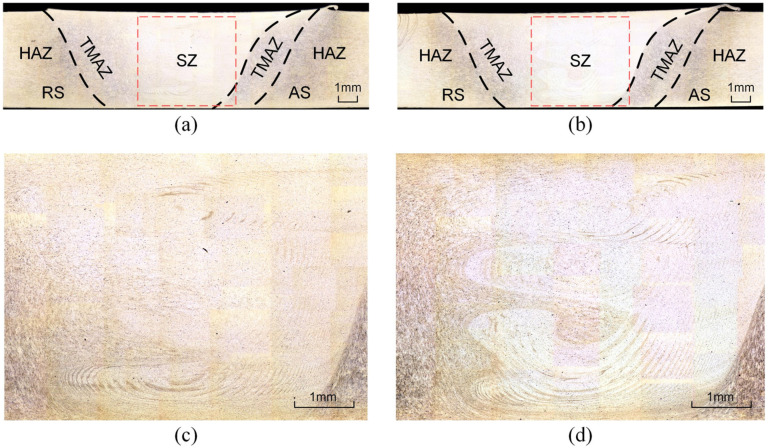
Metallographic microscopic image of 1200 rpm: (**a**) EE = 0.02 mm; (**b**) EE = 0.18 mm; (**c**) EE = 0.02 mm stirring zone; (**d**) EE = 0.18 mm stirring zone.

Metallographic observation and analysis can reveal the microstructural characteristics of materials, thereby evaluating their properties and quality. The weld seam cross-section of FSW includes the stirring zone (SZ), the thermo-mechanically affected zone (TMAZ), the heat-affected zone (HAZ), and the unaffected base material (BM). To further investigate the influence of vibration on the weld quality of RFSW, metallographic observation and analysis were conducted on the weld seam regions under different vibration conditions, with the spindle speed set at 1200 rpm, which was the most affected condition. After grinding and polishing, the specimens were corroded for 60 s using Keller’s reagent (2 mL HF + 3 mL HCl + 5 mL HNO3 + 90 mL H2O), and the microstructural images of the weld seam cross-section were observed using a laser confocal microscope, as shown in Figure 9.

Figure 9a,b show the micrographs at a spindle speed of 1200 rpm with eccentricity errors of 0.02 mm and 0.18 mm for the stirring tool, respectively. The primary differences observed between the two are within the stirring zone. The stirring zones of these images are magnified, as shown in Figure 9c,d. It is evident that with a larger eccentricity error, the layer structure formed in the stirring zone, under the influence of the stirring tool’s eccentricity and the robot’s end-effector vibration, becomes more complex. The metal material flow in the stirring area is more vigorous, leading to a more thorough mixing between materials, which in turn enhances the tensile strength of the weld area.

**Figure 10:**
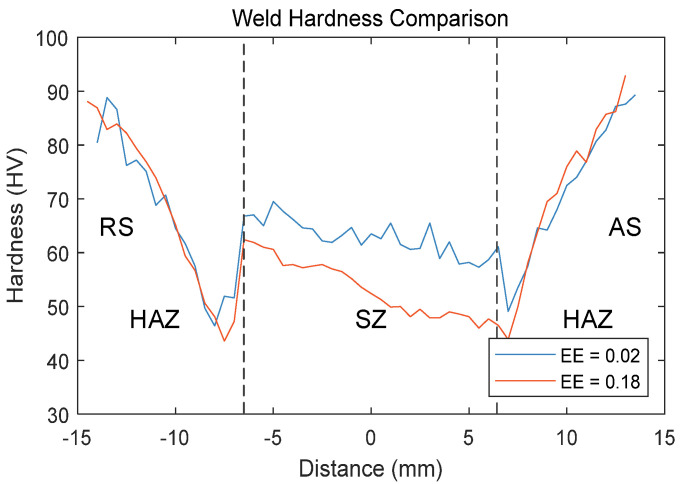
Weld surface hardness comparison.

The changes in grain size and dislocation density resulting from increased material flow are also reflected in the hardness variations within the weld region. The transverse hardness variations of the weld region for two sets of samples at a spindle speed of 1200 rpm were measured using a Vickers microhardness tester, as shown in Figure 10.

Both sets of samples exhibit a “W” shape distribution of hardness within the weld region. The base material shows the highest hardness, at around 90 HV, while the lowest hardness values are observed in the HAZ region on both sides of the weld, approximately 45 HV. Comparing two sets of hardness data, it is found that when the eccentricity error is large, the minimum hardness at the HAZ decreases, and the overall hardness of the SZ region also decreases. It can be seen that the increase in vibration intensity raises the welding temperature, thus reducing the surface quality of the weld seam.

**Figure 11:**
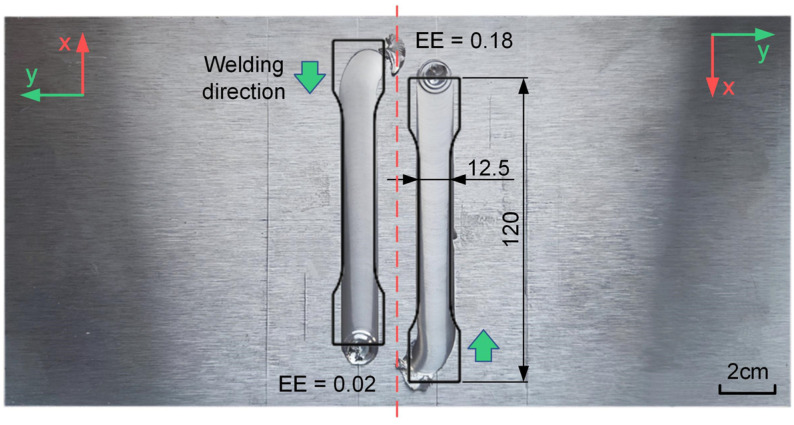
Welding experiments and longitudinal tensile specimens after changing pose.

**Figure 12:**
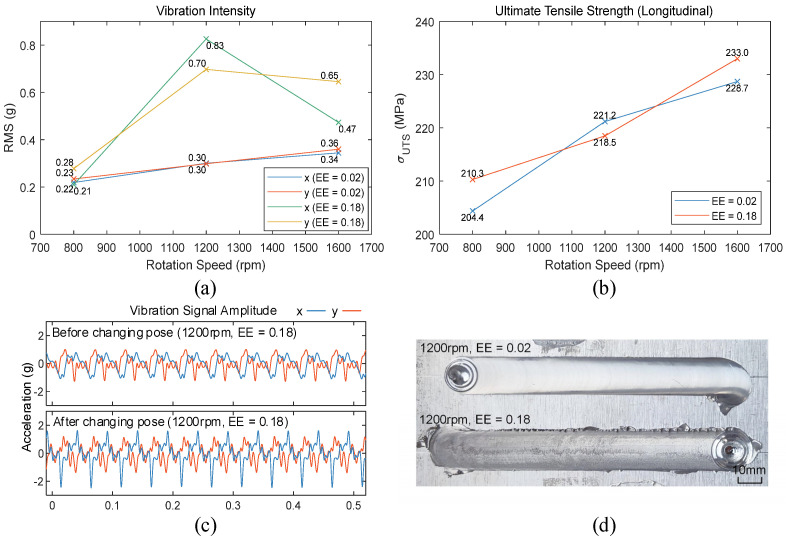
Welding experiment results after changing pose: (**a**) comparison of vibration intensity with different eccentricity errors; (**b**) comparison of longitudinal tensile strength with different eccentricity errors; (**c**) comparison of vibration signal amplitude with different poses; (**d**) weld surface with different eccentricity errors.

Based on prior experiments and existing research, it is known that the natural frequencies of a robot vary under different poses [28]. This study altered the welding poses, using the same stirring tool and welding parameters, and designed the welding experiments as shown in Figure 11. Initially, a stirring tool with a smaller eccentric error was used for the first welding along the −x direction indicated in the diagram. Subsequently, the plate was rotated 180 degrees, and a stirring tool with a larger eccentric error was used for the second welding in the same robot position. Welding experiments were conducted at spindle speeds of 800 rpm, 1200 rpm, and 1600 rpm. Vibration signals during welding were recorded, and longitudinal tensile tests on the weld zones were performed, with results presented in Figure 12.

Comparing Figure 12a with Figure 7 and Figure 12b with Figure 8b, it is evident that when the eccentric error of the stirring tool is small, both the vibration intensity at the robot’s end-effector and the longitudinal tensile strength of the weld are similar to those before the welding position was changed. However, when the eccentric error is larger, noticeable differences in vibration intensity and tensile strength are observed at 1200 rpm compared to before the change in welding position. The vibration intensity significantly increases, while the tensile strength decreases compared to when the eccentric error is smaller. The end-effector vibration signals at 1200 rpm, before and after changing the welding position, were subjected to a low-pass filter with a cutoff frequency of 200 Hz as shown in Figure 12c [32]. After changing the welding position, with a larger eccentric error, the amplitude of the robot end-effector vibration signal significantly increased, with peak acceleration values exceeding 2 g. Comparing the weld surface morphology as shown in Figure 12d, it is apparent that with a larger eccentric error, the surface quality of the weld decreases under intense vibrations. Thus, after changing the welding position, the robot’s natural frequency is closer to 20 Hz (1200 rpm), and at a spindle speed of 1200 rpm, a larger eccentric error causes severe resonance at the robot’s end-effector, leading to a decline in weld surface quality and mechanical properties.

Summarizing the two sets of vibration experiments in RFSW, it is evident that the primary cause of vibrations at the robot’s end-effector is the eccentric error of the stirring tool relative to the spindle. The intensity of these vibrations is influenced by the eccentric error, spindle speed, and the robot’s pose. The natural frequency of the FSW robot varies under different poses, and even within the same pose, the natural frequencies differ across different coordinate directions. In the mid to low spindle speed range, appropriate levels of vibration can enhance the mechanical properties of the welds, but excessive vibrations can deteriorate both the surface quality and mechanical properties of the welds.

### 3.2. Modal Analysis Results of the FSW Robot

The welding vibration experiments demonstrate that the robot’s pose significantly affects the vibration intensity during the RFSW process. Conducting modal analysis on the FSW robot to understand the variations in modal parameters is crucial for optimizing the welding process.

**Figure 13:**
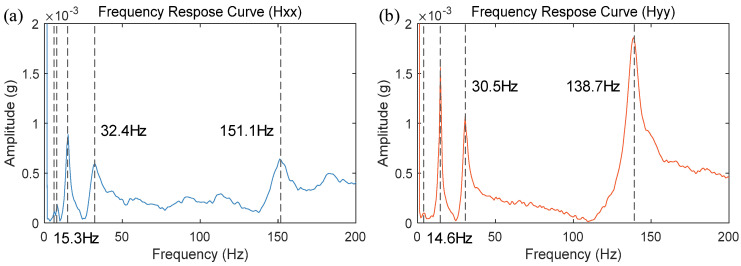
Frequency response curves: (**a**) Hxx; (**b**) Hyy.

The hammering impact test results at the robot’s working position are shown in Figure 13. The vibration response signals from different coordinate directions of the robot’s end-effector were then subjected to Fourier transform to obtain the frequency response curves at the current position. Since high-frequency components of the vibration response signals primarily originate from the end actuator and low-frequency components mainly from the robot’s body [40], the frequency response curves in the 0–200 Hz range are extracted as shown in Figure 13a,b.

**Figure 14:**
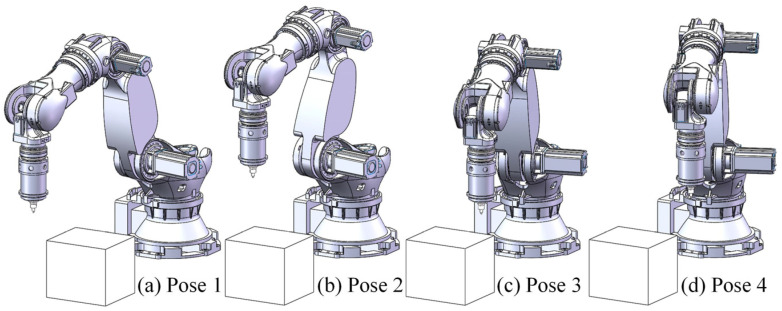
Robot poses near working position.

**Figure 15:**
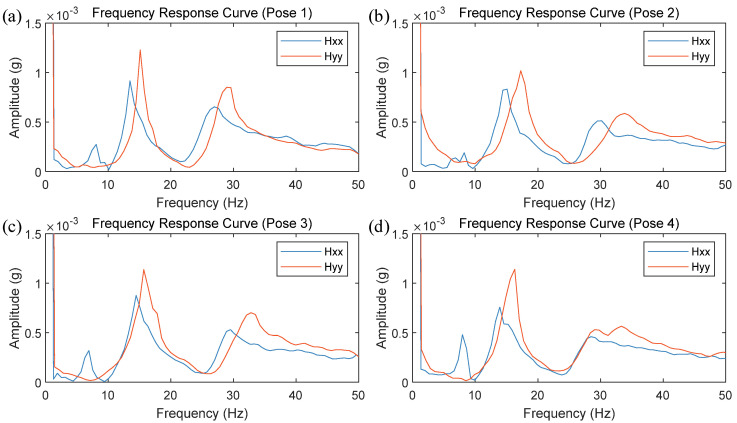
Frequency response curves of the robot in different poses (0–50 Hz): (**a**) pose 1; (**b**) pose 2; (**c**) pose 3; (**d**) pose 4.

Modal parameter identification of the frequency response curves revealed that there are five modal frequencies within the 0–200 Hz range for the FSW robot in its current position. Notably, the third and fourth orders modal frequencies fall within the typical rotational speed range used in FSW, significantly influencing the welding process. To further study the variation in modal parameters near the FSW robot’s working position, the robot’s end-effector posture was kept constant while measurements were taken at four different nearby locations using hammering testing. The robot’s poses are shown in Figure 14, and the obtained frequency response curves are displayed in Figure 15. The modal frequencies under different robot positions were determined from these frequency response curves, as shown in Table 2.

The modal analysis results for the FSW robot at different poses near the working location showed significant variations in modal frequencies depending on the robot’s pose and the coordinate direction. Near the working position, the third and fourth order modal frequencies ranged from 13 Hz (780 rpm) to 34 Hz (2040 rpm), covering nearly the entire commonly used speed range for the FSW process.

### 3.3. Simulation Verification of Active Vibration Avoidance Control Method

From the aforementioned experiments, it is evident that at mid to low spindle speeds, appropriate levels of vibration can facilitate material flow, enhance weld quality, and increase weld strength. Therefore, the RFSW process can be optimized by intentionally creating moderate eccentric errors or actively applying external vibrations to expand the process window and improve weld quality [13]. However, when the spindle rotational frequency matches or is close to one of the natural frequencies of the robot in its current pose, severe resonance occurs in the robot’s body, leading to a deterioration in weld quality.

Modal analysis of the FSW robot has revealed that within the workspace near the welding workbench, the modal frequencies at different robot poses essentially cover the entire commonly used speed range for FSW processes. Especially when performing complex welding tasks, such as welding intricate spatial curves, it is almost inevitable that the modal frequency at a certain robot pose will coincide with the spindle rotational frequency.

Welding parameters determine the heat input and material flow during the FSW process. If the robot’s natural frequencies across the entire working range are considered, the available process window becomes very narrow. Furthermore, without multi-axis linkage control via a positioner, once the weld piece’s position is fixed, the changes in the robot’s pose during the welding process are also fixed, and cannot be interrupted mid-process.

Therefore, to avoid resonance phenomena in the RFSW process simply, reliably, and accurately, this study investigates methods of vibration feedback control and explores variable speed welding techniques for FSW, then proposes an active vibration avoidance method for variable speed welding based on constant heat input as described in Section 2.2.

Based on the modal analysis results of the FSW robot at different poses, it is apparent that to achieve a significant change in modal frequency along the same coordinate direction without altering the end-effector’s posture, the robot’s end-effector needs to move a considerable distance. However, due to the spatial limitations of the actual workbench, it is not feasible to validate the variable speed welding method based on constant heat input experimentally within an effective workspace. Therefore, this paper employs simulation to conduct a qualitative analysis of the method. The simulation model is as described in Section 2.3

**Figure 16:**
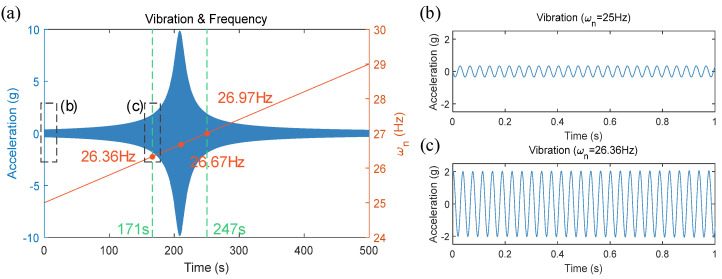
Welding process simulation without active vibration avoidance control: (**a**) vibration amplitude and modal frequency change curve; (**b**) vibration signal curve ($\omega_{n}$ = 25 Hz); (**c**) vibration signal curve ($\omega_{n}$ = 26.36 Hz).

Since the modal frequencies that significantly impact the FSW process are primarily the third and fourth modes, it is known from Table 2 that the fourth mode modal frequency of the robot varies around 26–30 Hz near the working position. Based on this, assume that during the welding process, the robot’s end-effector feeds from point A to point B along the x-axis, with points A and B being 1 m apart. The spindle speed during welding is 1600 rpm, and the welding speed is 2 mm/s. At point A, the modal frequency of the robot $\omega_{A}$ = 25 Hz, and at point B, $\omega_{B}$ = 29 Hz, with the modal frequency of the robot changing linearly from point A to point B. The variation of the robot’s modal frequency over time is shown by the red curve in Figure 16a.

At the start of the welding process, vibrations at the robot’s end-effector were minimal, with the modal frequency at 25 Hz. A 1 s segment of the vibration signal was captured and amplified as shown in Figure 16b, where the amplitude of the vibration signal was approximately 0.4 g. When the robot moved to a modal frequency of approximately 26.67 Hz (corresponding to a spindle speed of 1600 rpm), the amplitude of vibrations sharply increased, resulting in resonance which adversely affected both the robot itself and the welding quality.

Based on the welding vibration experimental results in Figure 12, it is assumed that a vibration signal amplitude exceeding 2 g indicates excessive vibration. (This value was not obtained through quantitative analysis experiments but rather assumed for simulation analysis.) Calculations revealed that the amplitude exceeds 2 g between approximately 171 s to 247 s, corresponding to frequencies from 26.36 Hz (about 1580 rpm) to 26.97 Hz (about 1620 rpm). A 1 s segment of the vibration signal at a modal frequency of 26.36 Hz is captured and amplified as shown in Figure 16c. The spindle speeds corresponding to these two frequencies differ by about 40 rpm, as indicated by the green dashed line in Figure 16a.

**Figure 17:**
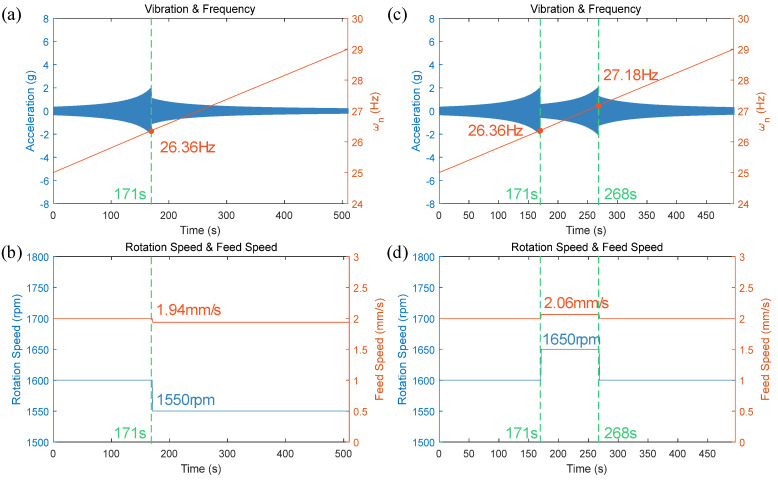
Simulation results of active vibration avoidance control: (**a**) vibration signal amplitude and modal frequency change curve 1; (**b**) rotation and welding speed change curve 1; (**c**) vibration signal amplitude and modal frequency change curve 2; (**d**) rotation and welding speed change curve 2.

Based on simulation results, the vibration signal intensity threshold was set at 2 g, and each spindle speed adjustment was set at 50 rpm to ensure that each adjustment crosses the resonance frequency range. Using the vibration feedback control process shown in Figure 3, the heat input balance equation as indicated in Equation (6), and the simplified dynamic model presented in Equation (7), the variable speed welding method was validated through simulation. The results are displayed in Figure 17.

Figure 17a,c respectively show the scenarios where the spindle speed is actively decreased and increased after the vibration signal amplitude exceeds the threshold. As shown in Figure 17a, when the spindle speed was actively reduced, the amplitude of the vibration signal decreased to below the threshold. According to the heat balance equation, the welding speed was synchronously reduced as depicted in Figure 17b. At this time, the spindle rotational frequency was less than the robot’s modal frequency. As the welding process continued, the robot’s modal frequency increased, thus keeping the vibration signal amplitude within the threshold. Figure 17c illustrates the scenario where the spindle speed was actively increased, leading to a reduction of the vibration signal amplitude to below the threshold, with the spindle rotational frequency still higher than the robot’s modal frequency. As per the heat balance equation, the welding speed was synchronously increased, as shown in Figure 17d. As the welding continued, the robot’s modal frequency increased, causing the vibration signal amplitude to exceed the threshold again. Since the spindle speed was higher than the set speed at this time, it was actively reduced, and the welding speed was also synchronously decreased, bringing the vibration signal amplitude back below the threshold until the end of welding.

Simulation results indicate that both reducing and increasing spindle speeds as part of the active variable speed welding method can avoid the modal frequencies of the FSW robot, effectively reducing the amplitude of end-effector vibration signals, while ensuring relatively stable welding parameters and consistent heat input during the welding process. However, since the changes in spindle speed are continuous, transient intense vibrations will occur when the rotational frequency crosses the robot’s modal frequency, suggesting that further refinements are needed for this method.

## 4. Conclusions

This paper investigates the vibration phenomenon, its effects, and optimization methods in RFSW processes. It is found that the eccentric error between the stirring tool and the spindle is the primary cause of vibrations during the RFSW process, while the intensity of these vibrations is influenced by both the spindle speed and the robot’s pose. Appropriate levels of vibration can enhance the mechanical properties of the welds; however, excessive vibrations degrade weld quality. Modal analysis of the FSW robot revealed that the third and fourth order modal frequencies significantly overlap with the commonly used speed range of the FSW process and have a substantial impact on the vibrations during welding. The simulation validation of the variable-speed welding active vibration avoidance control method using a simplified robot dynamic model shows that the method effectively avoids the robot’s modal frequencies. Under stable thermal input conditions during welding, excessive vibrations at the robot’s end due to resonance effects are prevented. The maximum amplitude of the vibration signal is reduced by approximately 80% compared to when the active vibration avoidance control method is not used. The simulation results are consistent with the expectations of the active vibration avoidance control method, theoretically demonstrating the feasibility of this approach.

There are still some limitations to this study. The simplified dynamics model does not fully replicate the characteristics of the robot, and further quantitative analysis would require developing a complete dynamics model of the robot or constructing an actual experimental platform. The simulation validation considered vibrations in only one direction, whereas the robot has different natural frequencies in different directions, necessitating consideration of more complex scenarios in practical applications. For further research, a comprehensive assessment of the factors influencing RFSW vibration is necessary. This entails investigating the vibration characteristics and weld quality under different welding speeds, materials, and welding types. Additionally, a cross-sectional evaluation of how vibration impacts FSW across various types of FSW equipment is warranted. This approach can leverage FSW’s vibration properties to enhance production efficiency and reduce maintenance costs.

## Figures and Tables

**Table 1:** Joint stiffness of FSW robot.

Joint	Stiffness (Nm/rad)
1	1.1676 × 10^6^
2	1.2731 × 10^8^
3	1.7187 × 10^6^
4	7.3227 × 10^5^
5	1.1011 × 10^6^
6	8.8932 × 10^5^

**Table 2:** The first four modal frequencies of the robot in different poses.

Pose	Direction	1st Mode (Hz)	2nd Mode (Hz)	3rd Mode (Hz)	4th Mode (Hz)
1	x	2.51	6.91	14.45	29.53
	y	3.56	5.70	15.69	32.80
2	x	4.04	8.09	13.48	26.95
	y	5.92	8.54	15.12	28.92
3	x	3.13	8.23	14.79	29.71
	y	3.32	8.63	17.26	33.86
4	x	3.31	7.95	13.91	28.49
	y	4.97	7.80	16.31	31.21

## Data Availability

Data are contained within the article.

## References

[1] Thomas W., Nicholas E., Needham J., Murch M., Temple-Smith P., Dawes C. (1991). Friction Stir Butt Welding. International Patent Application.

[2] Ilić A., Miletić I., Nikolić R.R., Marjanović V., Ulewicz R., Stojanović B., Ivanović L. (2020). Analysis of influence of the welding procedure on impact toughness of welded joints of the high-strength low-alloyed steels. Appl. Sci..

[3] Mendes N., Neto P., Loureiro A., Moreira A.P. (2016). Machines and control systems for friction stir welding: A review. Mater. Des..

[4] Ye X., Su Z., Dahari M., Su Y., Alsulami S.H., Aldhabani M.S., Abed A.M., Ali H.E., Bouzgarrou S.M. (2023). Hybrid modeling of mechanical properties and hardness of aluminum alloy 5083 and C100 Copper with various machine learning algorithms in friction stir welding. Structures.

[5] Gibson B.T., Lammlein D.H., Prater T.J., Longhurst W.R., Cox C.D., Ballun M.C., Dharmaraj K.J., Cook G.E., Strauss A.M. (2014). Friction stir welding: Process, automation, and control. J. Manuf. Process..

[6] Balaji V.A., Ajay G., Doss A.S.A., Schilberg D. (2023). Analysis of Robotic Arm for Friction Stir Welding Application. Lecture Notes in Mechanical Engineering.

[7] De Backer J., Bolmsjö G. (2014). Deflection model for robotic friction stir welding. Ind. Robot..

[8] Sun L.F., Fang L.J. (2018). An approximation method for stiffness calculation of robotic arms with hybrid open- and closed-loop kinematic chains. Adv. Mech. Eng..

[9] Bai Y., Liu H., Meng S., Ma Y., Wei Y., Yue W., Li G., Xiao J., Wang G., Ding Y. (2023). An Approach for Predicting and Compensating the End Deformation of a Heavy Load Robot for Friction Stir Welding. Mech. Mach. Sci..

[10] Li Z.W., Zhao H.H., Zhang X.C., Dong J.Y., Hu L., Gao H.M. (2023). Multi-parameter sensing of robotic friction stir welding based on laser circular scanning. J. Manuf. Process..

[11] Zhao J., Duan Y.X., Xie B.Y., Zhang Z.Q. (2021). FSW robot system dimensional optimization and trajectory planning based on soft stiffness indices. J. Manuf. Process..

[12] Xiao J.L., Wang M.L., Liu H.T., Liu S.J., Zhao H.H., Gao J.S. (2023). A constant plunge depth control strategy for robotic FSW based on online trajectory generation. Robot. Comput.-Integr. Manuf..

[13] Rahmi M., Abbasi M. (2017). Friction stir vibration welding process: Modified version of friction stir welding process. Int. J. Adv. Manuf. Technol..

[14] Bagheri B., Abbasi M., Dadaei M. (2020). Mechanical Behavior and Microstructure of AA6061-T6 Joints Made by Friction Stir Vibration Welding. J. Mater. Eng. Perform..

[15] Abbasi M., Bagheri B., Sharifi F. (2021). Simulation and experimental study of dynamic recrystallization process during friction stir vibration welding of magnesium alloys. Trans. Nonferrous Met. Soc. China.

[16] Xue F., He D.Q., Zhou H.B. (2022). Effect of Ultrasonic Vibration in Friction Stir Welding of 2219 Aluminum Alloy: An Effective Model for Predicting Weld Strength. Metals.

[17] Wu C.S., Wang T., Su H. (2022). Material flow velocity, strain and strain rate in ultrasonic vibration enhanced friction stir welding of dissimilar Al/Mg alloys. J. Manuf. Process..

[18] Tian W.H., Su H., Wu C.S. (2020). Effect of ultrasonic vibration on thermal and material flow behavior, microstructure and mechanical properties of friction stir welded Al/Cu joints. Int. J. Adv. Manuf. Technol..

[19] Guan W., Cui L., Liang H., Wang D.P., Huang Y.M., Li M., Li X.G. (2023). The response of force characteristic to weld-forming process in friction stir welding assisted by machine learning. Int. J. Mech. Sci..

[20] Shah L.H., Walbridge S., Gerlich A. (2019). Tool eccentricity in friction stir welding: A comprehensive review. Sci. Technol. Weld. Join..

[21] Mao Y.Q., Ke L.M., Liu F.C., Liu Q., Huang C.P., Xing L. (2014). Effect of tool pin eccentricity on microstructure and mechanical properties in friction stir welded 7075 aluminum alloy thick plate. Mater. Des..

[22] Panzer F., Werz M., Weihe S. (2018). Experimental investigation of the friction stir welding dynamics of 6000 series aluminum alloys. Prod. Eng..

[23] Luo H.T., Wang T.J., Fu J., Chen Z.C., Leng Y.Q. Analytical Kinematics and Working-condition Simulation for Friction Stir Welding (FSW) Robot. Proceedings of the 2015 Ieee International Conference on Information and Automation.

[24] Li M.J., Huang D.X., Han H.B., Yang X.J. (2023). Chatter Detection and Identification in High-Efficient Robotic Milling CFRP Composites Using Acoustic Emission Technique. Int. J. Precis. Eng. Manuf.-Green Technol..

[25] Ni H.P., Ji S., Ye Y.X. (2022). Redundant Posture Optimization for 6R Robotic Milling Based on Piecewise-Global-Optimization-Strategy Considering Stiffness, Singularity and Joint-Limit. Symmetry.

[26] Nguyen V., Johnson J., Melkote S. (2020). Active vibration suppression in robotic milling using optimal control. Int. J. Mach. Tools Manuf..

[27] Xin S., Peng F., Tang X., Wu J., Sun Z., Yan R. (2023). A joint wearable structural reinforcing device for vibration suppression in robotic milling. MM Sci. J..

[28] Chen C., Peng F., Yan R., Tang X., Li Y., Fan Z. (2020). Rapid prediction of posture-dependent FRF of the tool tip in robotic milling. Robot. Comput.-Integr. Manuf..

[29] Nguyen V., Cvitanic T., Melkote S. (2019). Data-driven modeling of the modal properties of a six-degrees-of-freedom industrial robot and its application to robotic milling. J. Manuf. Sci. Eng..

[30] Futami S., Kyura N., Hara S. (1983). Vibration absorption control of industrial robots by acceleration feedback. IEEE Trans. Ind. Electron..

[31] Dai Y., Jia B., Zhang J., Cao G., Xia G. (2019). Motion Control of Milling Robot Based on Vibration Feedback. J. Tianjin Univ. Sci. Technol..

[32] Zong G., Kang C., Chen S., Jiang X. (2024). Optimization of Installation Position for Complex Space Curve Weldments in Robotic Friction Stir Welding Based on Dynamic Dual Particle Swarm Optimization. Processes.

[33] Xu W., Liu J., Zhu H. (2010). Numerical simulation of thermal field of friction stir welded 2219 aluminum alloy thick plate. Trans. China Weld. Inst..

[34] Yin P., Zhang R., Xiong J., Zhao K., Li J. (2012). An Effective Numerical Simulation of Temperature Distribution of Friction Stir Welding in Quasi-Steady-State. J. Northwestern Polytech. Univ..

[35] Chang C., Lee C., Huang J. (2004). Relationship between grain size and Zener–Holloman parameter during friction stir processing in AZ31 Mg alloys. Scr. Mater..

[36] Fang Q., Li C., Fei S., Meng T. (2016). Stability analysis of robot boring system. Acta Aeronaut. Et Astronaut. Sin..

[37] Chen S., Zong G., Kang C., Jiang X. (2024). Digital Twin Virtual Welding Approach of Robotic Friction Stir Welding Based on Co-Simulation of FEA Model and Robotic Model. Sensors.

[38] Jain P.H., Bhosle S.P. (2022). Analysis of vibration signals caused by ball bearing defects using time-domain statistical indicators. Int. J. Adv. Technol. Eng. Explor..

[39] Dorbane A., Ayoub G., Mansoor B., Hamade R., Imad A. (2017). Effect of temperature on microstructure and fracture mechanisms in friction stir welded Al6061 joints. J. Mater. Eng. Perform..

[40] Cordes M., Hintze W., Altintas Y. (2019). Chatter stability in robotic milling. Robot. Comput.-Integr. Manuf..

